# Apigenin Attenuates Hippocampal Microglial Activation and Restores Cognitive Function in Methotrexate-Treated Rats: Targeting the miR-15a/ROCK-1/ERK1/2 Pathway

**DOI:** 10.1007/s12035-023-03299-7

**Published:** 2023-03-21

**Authors:** Mohamed Taha, Omar Mohsen Eldemerdash, Ismail Mohamed Elshaffei, Einas Mohamed Yousef, Ayman S. Soliman, Mahmoud Ahmed Senousy

**Affiliations:** 1 Department of Biochemistry, Faculty of Pharmacy, Cairo University, Kasr El Ainy st., Cairo, 11562 Egypt; 2 Department of Biochemistry, Faculty of Pharmacy, Misr International University (MIU), KM 28 Cairo, Ismailia Road, Cairo, 44971 Egypt; 3 Department of Histology and Cell Biology, Faculty of Medicine, Menoufia University, Shibin El Kom, Egypt; 4 Medical Physiology Department, Faculty of Medicine, Beni-Suef University, Beni-Suef, Egypt; 5 Department of Biochemistry, Faculty of Pharmacy and Drug Technology, Egyptian Chinese University, Cairo, 11786 Egypt

**Keywords:** Apigenin, Methotrexate, miR-15a, Microglial activation, Iba-1, ROCK-1

## Abstract

Microglial activation underpins the methotrexate (MTX)-induced neurotoxicity; however, the precise mechanism remains unclear. This study appraised the potential impact of apigenin (Api), a neuroprotective flavonoid, in MTX-induced neurotoxicity in rats in terms of microglial activation through targeting the miR-15a/Rho-associated protein kinase-1 (ROCK-1)/extracellular signal-regulated kinase 1/2 (ERK1/2) pathway. Male Sprague Dawley rats were randomly divided into 4 groups: Normal control (saline i.p. daily and i.v. on days 8 and 15); Api control (20 mg/kg, p.o.) daily for 30 days; MTX-alone (75 mg/kg, i.v.) on days 8 and 15, then four i.p. injections of leucovorin (LCV): 6 mg/kg after 18 h, then three doses (3 mg/kg) every 8 h post-MTX; and Api co-treated (20 mg/kg/day, p.o.) throughout the model for 30 days, with administration of MTX and LCV as in group 3. MTX administration elevated hippocampal ionized calcium-binding adaptor protein-1 (Iba-1) immunostaining, indicating microglial activation. This was accompanied by neuroinflammation, oxidative stress, and enhanced apoptosis manifested by elevated hippocampal interleukin-1β, malondialdehyde, and caspase-3, and decreased reduced glutathione levels. Concurrently, abated miR-15a expression, overexpression of its target ROCK-1, diminished downstream ERK1/2 and cAMP response element-binding protein (CREB) phosphorylation, and decreased hippocampal brain-derived neurotrophic factor (BDNF) levels were observed. Api mitigated the MTX-induced neurotoxicity by reversing the biochemical, histopathological, and behavioral derangements tested by novel object recognition and Morris water maze tests. Conclusively, Api lessens MTX-induced neuroinflammation, oxidative stress, and apoptosis and boosts cognitive function through inhibiting microglial activation via modulating the miR-15a/ROCK-1/ERK1/2/CREB/BDNF pathway.

**Graphical Abstract:**

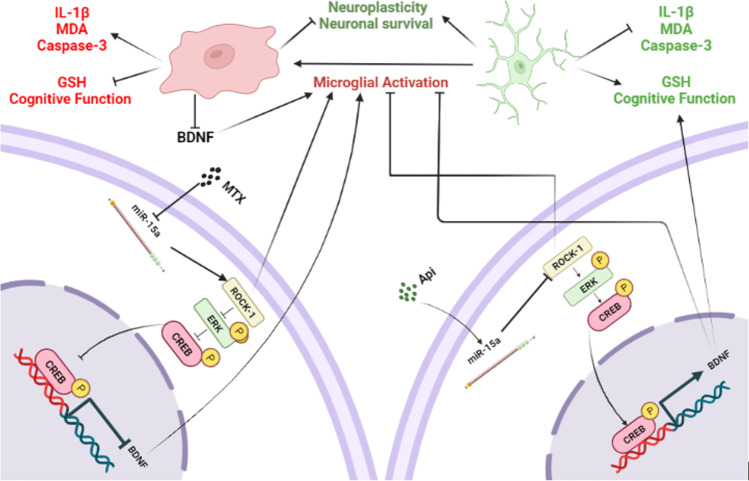

***Graphical abstract showing the effects of methotrexate and apigenin co-treatment in MTX-induced neurotoxicity model.***

*On the left, methotrexate (MTX) administration to rats resulted in hippocampal miR-15a downregulation, which triggered an enhanced expression of its target ROCK-1, consequently inhibiting the downstream ERK1/2/CREB/BDNF pathway, instigating a state of microglial activation, neuroinflammation, oxidative stress, and apoptosis. On the other hand, apigenin (Api) co-treatment restored miR-15a, inhibited ROCK-1 expression, and activated the ERK1/2/CREB/BDNF pathway, leading to diminished hippocampal microglial activation, neuroinflammation, and apoptosis, and restoration of the redox balance, along with improvement in memory and cognitive function of the MTX-treated rats.*

## Introduction

Methotrexate (MTX) is one of the most routinely employed chemotherapeutic agents in acute lymphoblastic leukemia (ALL) and other cancers; however, its use has been linked to an increased incidence of neurotoxicity in about 9–53% of patients [1, 2]. This is evident as cognitive impairment, memory problems, and motor dysfunction which appear in 30–60% of pediatric cancer survivors who have undergone chemotherapy [2, 3]. The severity of MTX-induced neurotoxicity is multifactorial, with many predisposing factors influencing its severity, including age, dose, route of administration, and exposure to radiation [3]. Although several theories have been proposed to contemplate the neurotoxic actions of MTX, the exact mechanism is still unknown.

Microglial activation, neuroinflammation, and activation of apoptosis are among the mechanisms expected to be involved in MTX-induced neurotoxicity, ultimately culminating in loss of spatial working memory and cognitive impairments [4]. Indeed, Wen et al. [5] reported that 50% of MTX-treated rats showed a substantial decrease in hippocampal neurogenesis, cell proliferation as well as increased microglial activation and apoptosis compared to controls [5]. Peculiarly, MTX was reported to activate the microglia, prompting escalated inflammatory response [6]. The microglia are neuronal macrophages, which release chemokines and cytokines, activate apoptosis, inhibit cell proliferation, and maturation [7]. Persistent activation of the inflammatory microglia can cause neurotoxicity by dysregulation of astrocytes and oligodendrocytes, decreasing neuronal myelination, survival, and neurogenesis, and increasing neuroinflammation, finally precipitating cognitive impairment and memory deficits [6, 8]. Recently, MTX was shown to induce chronic microglial activation [6, 9]. However, the exact molecular pathway is still unclear.

Recent studies demonstrate the crucial role of Rho-associated protein kinase-1 (ROCK-1) in various neurodegenerative diseases [10, 11]. ROCK-1 plays a critical role in microglial inflammatory cytokine synthesis and release via controlling microglial activation [12]. Inhibition of ROCK-1 activity was reported to affect the microglial phenotype, which might contribute to the neuroprotective effects noted following ROCK-1 inhibition in various models [13–15]. Furthermore, ROCK-1 induction increases neuroinflammation not only via microglial activation but also by its downregulatory effect on extracellular signal-regulated kinase 1/2 (ERK1/2) and the downstream mediators cAMP response element-binding protein (CREB)/brain-derived neurotrophic factor (BDNF) [16]. These data emphasize the possible role of the ROCK-1/ERK1/2 pathway in microglial activation, but whether this extends to the MTX-induced neuroinflammation has not been previously tackled.

Recently, microRNAs (miRNAs) have been interconnected with memory regulation and neurogenesis. Among various miRNAs, miR-15a was demonstrated to be downregulated in Alzheimer’s disease (AD) brains; its overexpression has been proven to improve spatial memory and cognitive performance in AD mice models [17]. A recent study has shown that miR-15a targets and represses the ROCK-1 gene in hippocampus neurons, and that its increased expression boosted cell survival while decreasing apoptosis [18]. However, the effect of MTX on miR-15a and its downstream ROCK-1 as well as their impact on microglial activation is poorly elucidated.

Apigenin (Api) is a flavonoid that occurs naturally in a range of fruits and vegetables, including chamomile flowers, garlic, parsley, guava, onion, tea, and citrus fruits [19]. Among the many recognized biological effects of Api are its antioxidant and anti-inflammatory properties as well as its ability to fight cancer and other serious diseases [20]. The neuroprotective effect of Api was recently identified in a rat model of pentylenetetrazol-induced cognitive impairment [19] as well as in vitro models of neuroinflammation associated with AD [21]. However, the full neuroprotective and restorative mechanisms of Api are still to be investigated.

With this scenario, we hypothesized that Api has the premise as a beneficial candidate for co-treatment with MTX to mitigate its neurotoxic effects and behavioral and memory derangements. Thereby, this study was designated to investigate the potential involvement of the miR-15a/ROCK-1/ERK1/2 pathway in MTX-induced neurotoxicity in rats from the perspective of microglial activation. Specifically, the neuroprotective effect of Api was examined in terms of its impact on miR-15a/ROCK-1/ERK1/2/CREB/BDNF pathway, microglial activation, neuroinflammation, oxidative stress, and apoptosis. Microglial activation was examined using the immunofluorescence staining of ionized calcium-binding adaptor protein-1 (Iba-1).

## Materials and Methods

### Experimental Animals

Sixty male Sprague Dawley rats, weighing 150–200 g and aged 4–5 weeks, were obtained. The animals were kept in propylene cages (5 per cage) in a controlled environment at Misr International University’s animal house, with a consistent temperature (25 °C ± 2 °C), humidity (60 ± 10%), and a 12/12-h light/dark cycle as well as unrestricted access to water and pellet diet.

The Research Ethics Committee for Experimental and Clinical Studies, Faculty of Pharmacy, Cairo University, Cairo, Egypt, authorized all animal procedures and experimental protocols (Permit Number: BC2944). The animals were housed according to the US National Institutes of Health publication Guide for Care and Use of Laboratory Animals (No. 85–23, revised 2011). All attempts were made to reduce the number of animals used and minimize animal suffering.

### Drugs and Chemicals

MTX was procured from Mylan S.A.S., France as 50 mg/vial ready for intravenous (i.v.) administration. Api (50 mg/capsule) was purchased from Swanson, USA; each capsule was dissolved into 10 ml of 0.5% carboxymethyl cellulose (CMC) immediately before oral administration. Leucovorin (LCV) (Calcifolinon^®^, 50 mg/vial) was supplied from GPI, Egypt. CMC sodium salt was purchased from TopChem, Egypt.

### Experimental Design

During the design phase of the experiment, we conducted a pilot study in which we found that the administration of four i.p. doses of LCV following MTX administration is vital to maintain the rats alive by lessening the MTX-induced fatal diarrhea and weight loss. The significant mortality rate in the pilot study led us to start the study model with 15 rats/group to ensure sufficient samples at the end of the experiment.

**Fig. 1:**
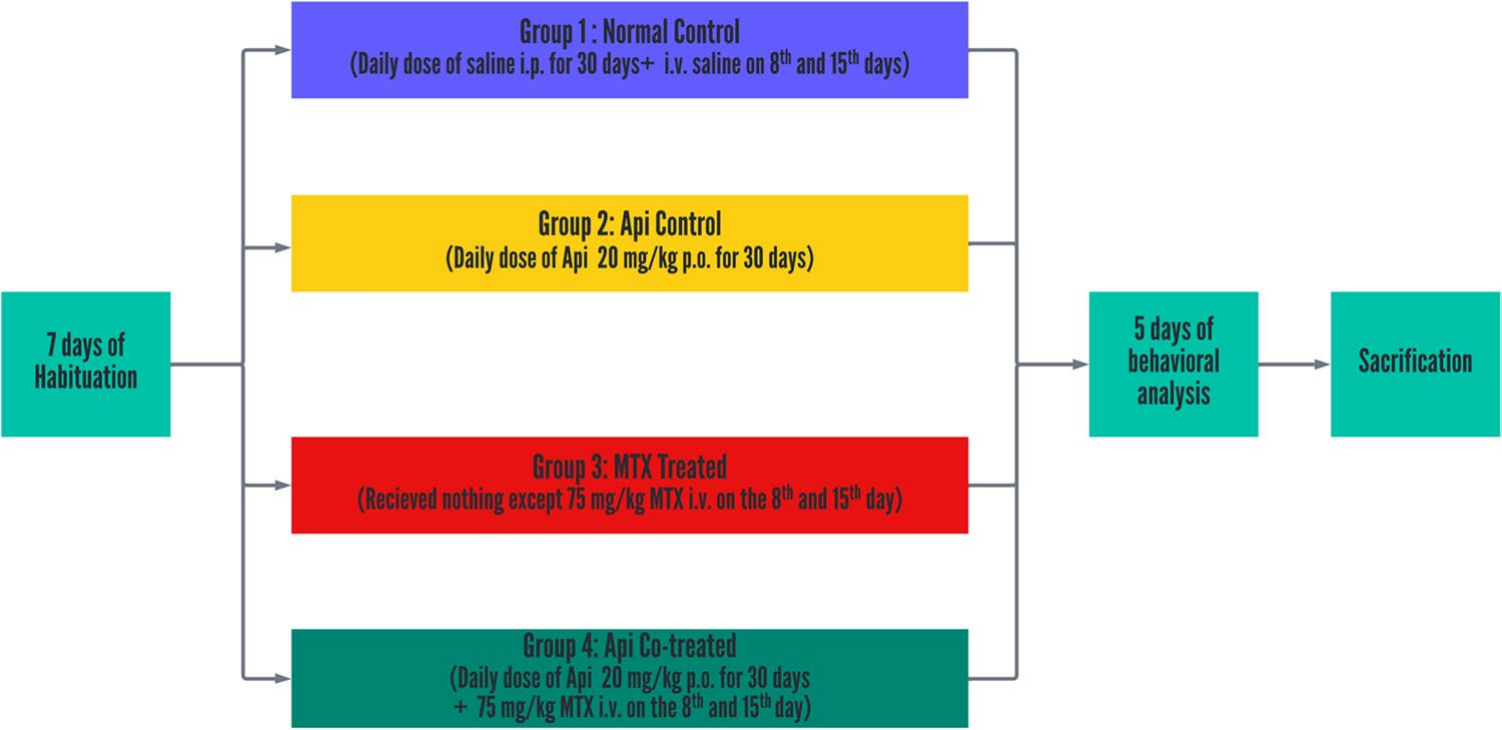
Experimental design

Rats were randomly divided into four groups of 15 rats each. The group size was calculated using power analysis (power = 0.9, *α* = 0.05). The rats had been acclimated to the new environment for 7 days. The experimental design is displayed in Fig. 1 as follows:

- Group 1 (Normal control): rats received saline intraperitoneally daily for 30 days and intravenously on the 8^th^ and 15^th^ days of the model.
- Group 2 (Api control): rats received a daily dose of Api (20 mg/kg, p.o.) dissolved in 0.5% CMC for 30 days [19].
- Group 3 (MTX only): rats received MTX (75 mg/kg, i.v.) on the 8^th^ and 15^th^ days of the model [22], afterwards they received four i.p. injections of LCV: the first dose was 6 mg/kg after 18 h, and the following three doses were 3 mg/kg after 26, 42, and 50 h of MTX administration [22–24].
- Group 4 (Api co-treated): Api (20 mg/kg/day, p.o.) was administered daily to rats throughout the study for 30 days, with administration of MTX (75 mg/kg, i.v.) on days 8 and 15, followed by LCV injections as in group 3.

All doses were selected based on previously published data. All models studying MTX-induced neurotoxicity were 30 days long in which MTX (75 mg/kg, i.v.) is administrated on both the 8^th^ and 15^th^ days of the model [22, 24]. Api was administered in 20 mg/kg, p.o. based on a previous trial which supported the use of 20 mg/kg over the use of 10 mg/kg in attenuating cognitive impairment and CREB/BDNF upregulation in kindled mice [19]. Furthermore, Api was used in the same dose as hepatoprotective and neuroprotective flavonoid in bile duct ligation [25] and in nephropathy [26]. LCV doses were chosen based on previous studies [22–24] and our pilot study.

Assessments of the rats’ memory and cognitive functions were carried out after 30 days for all groups (10 rats per group). Twenty-four hours following the behavioral testing, sacrification was performed by cervical dislocation after being anesthetized with 3% isoflurane inhalation. Right away, brains were dissected and quickly rinsed in ice-cold saline. Four whole brains from each group were fixed in 10% neutral buffered formalin, and then sent to the pathology lab for histopathological and immunohistochemical examination. The hippocampi of the remaining six brains were dissected from each side of the brain and frozen at − 80 °C until the biochemical parameters were tested. All assessments were conducted by blinded investigators.

### Behavioral Tests

Novel object recognition (NOR) and Morris water maze (MWM) were selected due to their effectiveness in assessing memory and cognitive function, their availability in the animal house, and the availability of ANY-maze software for automatic analysis. NOR is a frequently utilized behavioral examination for spatial memory that investigates the short- and long-term spatial memory associated with the hippocampus [27, 28]. Furthermore, MWM is utilized to identify spatial learning and memory in hippocampal-affected animals [28].

#### NOR Test

At the end of the model, NOR was carried out in order to evaluate the rats’ capacity to recall the previously studied object and identify the new one. Each animal was trained for 5 min in an empty open field arena (1 m × 1 m × 0.5 m) the day before the test. On the day of the examination, every rat participated in two trials: a familiarization trial and a choice trial. Both trials were monitored, recorded, and analyzed with the help of ANY-maze software. After each test, the arena and the objects in it were disinfected with ethanol at a concentration of 20% to eradicate any trace of a scent.

During the familiarization phase of the experiment, each rat was exposed to two of the same objects. After allowing each rat to investigate the items for a period of 3 min, they were removed from the arena and placed in a separate cage for a period of 15 min. After this period of time had passed, one of the objects that were being used in the choice experiment was switched out for a brand-new object. During the choice trial, each rat was taken back to the arena and presented with one familiar object and one unfamiliar object. They were given 3 min to investigate the items before being taken out of the arena and returned to their regular cages. The amount of time spent investigating each object while the rat’s head was pointed toward the thing from a distance of less than 2 cm away from the object was logged [23].

#### MWM Test

The MWM test was used to determine the rats’ spatial learning ability. The rats began the training phase of the MWM 1 day after the model ended, with 12 training trials in which each rat received four training trials per day for three consecutive days before receiving a probe trial [29]. During the training phase, the platform was positioned in the pool’s south-east (SE) quadrant. Each rat began a 1-min trial from one of four different starting locations, and each rat received four different trials from each starting position on a daily basis, as shown in Table 1. Each trial was limited to 1 min in duration, and if the rat did not reach the platform, it was directed to it for 15 s before being returned to its cage. The rats received 1 h of rest in between trials. On the fourth day, the rats had the probe test in which the platform was removed. The rats started from the NW position for 1 min. The quadrant time percentage, path efficiency, and escape latency were detected. ANY-maze software was used to record and analyze the trials and the probe trial.

**Table 1:** Morris water maze start positions

Day	Trial 1	Trial 2	Trial 3	Trial 4
Day 1	N	W	SW	NE
Day 2	SW	N	NE	W
Day 3	NE	SW	W	N
Day 4	Probe trial started at NW			

*N*, North; *W*, West; *SW*, South-west; *NE*, North-east; *NW*, North-west

### miRNA Selection

We relied on a bioinformatics approach using the miRTargetLink 2.0 interactive tool (https://ccb-compute.cs.uni-saarland.de/mirtargetlink2/network/bf99b12a-cd13-453e-bcc1-aa4226ac943e) as well as on previously reported experimental validation to confirm the interaction of miR-15a and ROCK-1 [18]. We also employed the Human MicroRNA Disease Database (HMDD) (https://www.cuilab.cn/hmdd) to detect the association between the selected miRNA and neurological disorders. Indeed, the association of miR-15a with AD, epilepsy, and multiple sclerosis was recorded in the database.

### Biochemical Assays

Homogenization of rat brain hippocampal tissue was processed as directed by the manufacturer using cold phosphate-buffered saline, and a 10% tissue homogenate was prepared.

#### Colorimetric Assay

Reduced glutathione (GSH) and malondialdehyde (MDA) concentrations were determined using colorimetric kits manufactured by Biodignostics^®^, Giza, Egypt (Cat. No. GR 25 11, MD 25 29). All assays were conducted in accordance with the manufacturer’s specifications in a 10% hippocampal tissue homogenate.

#### Enzyme-Linked Immunosorbent Assay (ELISA)

For the analysis of total CREB (t-CREB), phosphorylated CREB (p-CREB), BDNF, interleukin (IL)-1β, and caspase-3, rat ELISA kits were used. The total CREB and p-CREB ELISA kits were purchased by FineTest^®^, China (Cat. No: ER0914) and AFG Bioscience^®^, USA (Cat. No: EK742597), respectively. BDNF and IL-1β ELISA kits (Cat. No: E-EL-R2084 for BDNF and Cat. No. E-EL-R0267 for IL-1β) were manufactured by Elabscience^®^, USA. The caspase-3 ELISA kit was obtained from Cusabio^®^, USA (Cat. No: CSB-E07264r). Following the manufacturer’s instructions, all tests were carried out on a 10% hippocampal tissue homogenate using the Sandwich ELISA technique.

Data of ELISA and colorimetric assays are expressed per mg protein as measured using Bradford method.

#### Reverse Transcriptase-Quantitative Polymerase Chain Reaction (RT-qPCR)

Total RNA was deduced from lysates of rat brain hippocampal tissue using Direct-zol RNA Miniprep Plus (Cat. No. R2072, Zymo Research Crop, USA), according to the manufacturer’s protocol. A Beckman dual spectrophotometer (USA) was used to determine the concentration and purity of the extracted RNA. The optical density of isolated RNA was determined at 260 nm and 280 nm to guarantee its purity. The entire RNA was reverse transcribed into complementary DNA (cDNA). For analyzing the expression levels of miR-15a and ROCK-1, we utilized Thermo Fisher Scientific’s Superscript IV One-Step RT-PCR kit (Cat. No. 12594100, USA) for reverse transcription and real-time PCR in a single step using Step-One Applied Biosystem PCR apparatus, USA according to the manufacturer’s protocol. The primers’ sequences of the studied genes and the reference genes are mentioned in Table 2.

**Table 2:** Sequences of primers used in the study

Gene	Accession number	Primer sequence (5’-3’)
*miR-15a*	XR_005648635.1	F: GCCGAGTAGCAGCACACATAA
		R: CAGTGCGTGTCGTGGAGT
*RNU6*	XR_006711274.1	F: GCTTCGGCAGCACATATACTAAA
		R: CGCTTCACGAATTTGCGTGTCAT
*ROCK-1*	NR_171200.1	F: AATCTTCCAGTTGGTTCTGCCT
		R: CTCTATTTGGTACAGAAAGCCAACC
*GAPDH*	NM_001394060.2	F: CCTTCTCCATGGTGGTGAAGA
		R: CACCATCTTCCAGGAGCGAG

The results of RT-qPCR were translated into cycle threshold (Ct) values. The Ct values for the studied genes miR-15a and ROCK-1 were determined and corrected using the delta-delta Ct method normalizing to the housekeeping genes RNU6 and GAPDH, respectively. We determined the fold change of each gene by taking 2^−∆∆Ct^.

#### Western Blotting

Total and phosphorylated ERK1/2 (p-ERK1/2) levels in hippocampal tissues were measured using a western blot. The ReadyPrep^®^ complete protein extraction kit from BIO-Rad Inc., USA (Cat. No. 163–2086) was used to extract protein. BIO Basic Inc., Canada’s Bradford Protein Assay Kit (Cat. No. SK3041) was used to assess protein concentration in each sample. Each 20 µg protein sample was then loaded with 2 × Laemmli sample buffer containing 4% sodium dodecyl sulphate (SDS), 10% 2-mercaptoethanol, 20% glycerol, 0.004% bromophenol blue, and 0.125 M Tris HCl, and the pH was adjusted to 6.8. The mixture was heated at 95 °C for 5 min before polyacrylamide gel electrophoresis to denature the protein. Polyacrylamide gels were made with Bio-Rad Laboratories Inc. TGX Stain-Free™ FastCast™ Acrylamide Kit (SDS-PAGE) (Cat. No: 161–0181). The SDS-PAGE was made as directed. From bottom to top, the gel was built in a transfer sandwich (filter paper, PVDF membrane, gel, and filter paper). The sandwich was placed in 1 × transfer buffer (25 mM Tris, 190 mM glycine, and 20% methanol). Next, protein bands were transferred from the gel to the membrane at 25 V for 7 min using a Bio-Rad Trans-Blot Turbo. For 1 h, the membrane was occluded in Tris-buffered saline (TBS) blocking buffer components: 3% bovine serum albumin, 0.1% Tween 20, 20 mM Tris, 150 mM NaCl, pH 7.5. We used p-ERK1/2 and total ERK1/2 primary antibodies [diluted 1:500 in Tris-buffered saline and Tween (TBST)]. Each primary antibody was incubated overnight at 4 °C. The blot needed 3–5 TBST rinses over 5 min. The target protein was incubated in the horseradish peroxidase (HRP)-conjugated secondary antibody solution for 1 h at room temperature as previously described [30].

The chemiluminescent substrate (Clarity™ Western ECL substrate Bio-Rad Cat. No: 170–5060) was added to the blot in accordance with the manufacturer’s instructions. CCD camera-based imagers recorded the chemiluminescent signals. ChemiDoc MP imager band intensities were compared to β-actin (housekeeping protein) using image analysis tools.

### Histopathology and Immunohistochemistry

After being cleaned and fixed in 10% neutral buffered formalin for 72 h, samples were washed with xylene, infiltrated, and then embedded in Paraplast tissue embedding media after being trimmed and treated with different grades of ethanol. Hippocampal regions in distinct samples were demonstrated using rotatory microtome sections of 4-μm-thick sagittal brain.

The general morphological examination was performed using H&E staining. In Dentate gyrus (DG) and Cornu ammonis (CA3), Nissl staining with toluidine blue staining was used to identify damaged and intact neurons. The Handbook of Histopathological and Histochemical Techniques was followed for all fixation and staining procedures [31].

To perform immunohistochemistry, preparation of 5-μm-thick paraffin embedded tissue sections was carried out in line with the manufacturer’s instructions. 0.3% H_2_O_2_ was applied to deparaffinized tissue sections for 20 min. Afterwards, anti-Iba-1 antibody was used to treat the brain samples overnight at 4 °C (ab108539-Abcam — 1:100). After washing with phosphate-buffered saline, tissue sections were incubated for 20 min with the HRP Envision kit (DAKO) secondary antibody. The sample was then washed and incubated for 15 min with diaminobenzidine. The sample was then dehydrated, cleaned in xylene, and covered for microscopic examination.

According to Abbas et al. [32], Iba-1 immuno-expression levels in immunohistochemically stained sections were determined by the image analyzer computer system; Image J software (Version 1.53 K, National Institute of Health, USA) using six non-overlapping fields from each sample [32]. Furthermore, the number of intact neurons in both DG and CA3 was assessed using the same software [33]. The Leica Application module for histological analysis and a full HD microscopic imaging system (Leica Microsystems GmbH, Germany) were used for all light microscopic exams and data acquisition.

### Statistical Analysis

G*Power software was used to calculate the sample size per group using power analysis (power = 0.9, α = 0.05). The mean and standard deviation (SD) were utilized to mathematically express the data. The tests of Shapiro–Wilk and Kolmogorov–Smirnov were used to determine whether the variables in this study followed a normal distribution. One-way ANOVA with Tukey’s post hoc test was performed to compare the results of the various groups, except for the training and probe escape latency where two-way ANOVA was used. The Dixon *Q* test was utilized to identify the outliers. Mead’s “Resource Equation” was applied to evaluate whether the sample size is statistically sufficient. All statistical analyses were carried out with the help of the GraphPad Prism 9.0.0 statistical analysis program (121). The correlations between various parameters were analyzed using Pearson’s correlation. For each test, a *P* value of less than 0.05 was used to show statistical significance.

## Results

### Api Restores Cognitive Function in MTX-Treated Rats

**Fig. 2:**
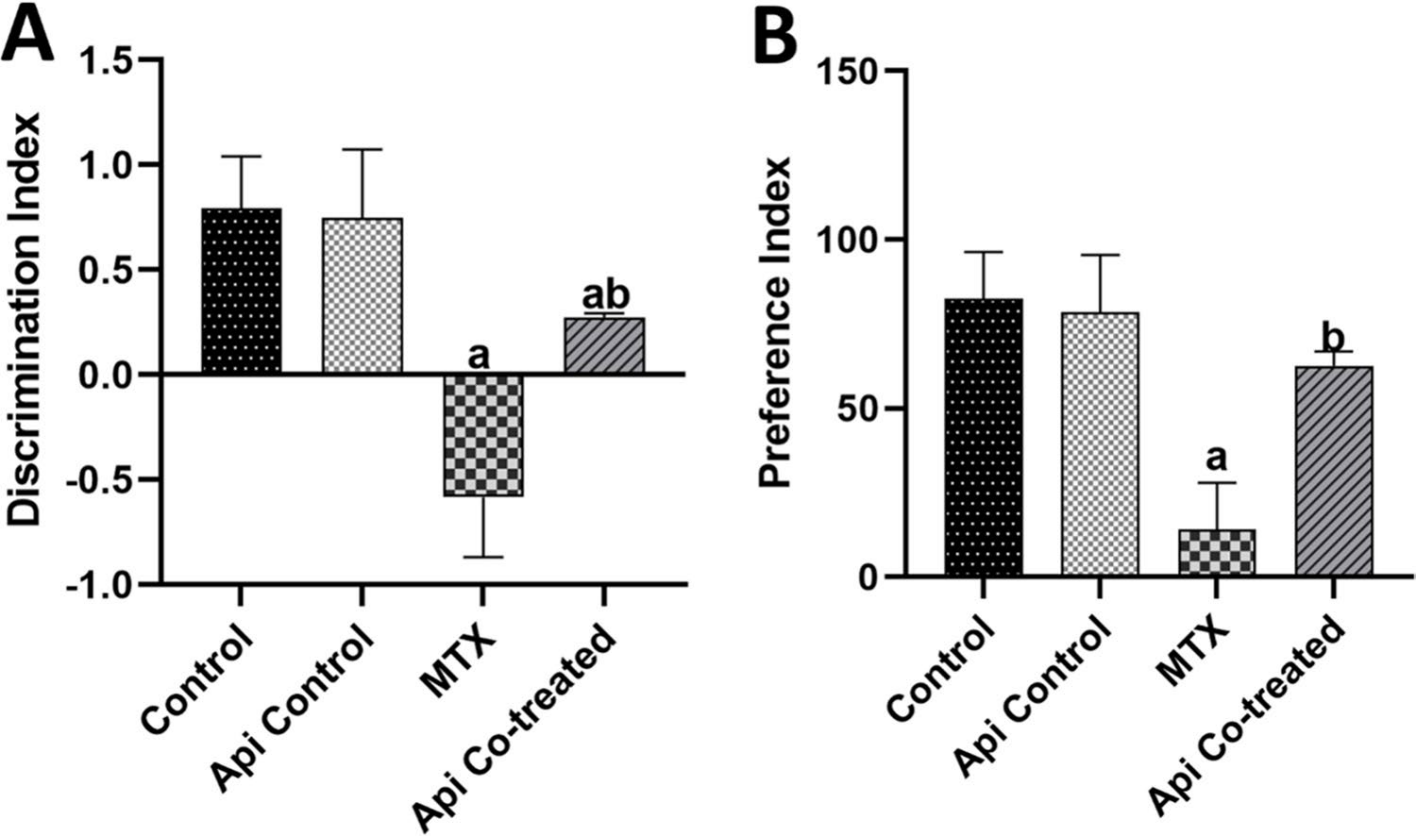
Effects of MTX and Api co-treatment on cognitive function (novel object recognition test). Data are expressed as mean ± SD, *n* = 10. (**a**) significant difference from control, (**b**) significant difference from MTX. Significance level was set at *P* < 0.05. One-way ANOVA test and Tukey’s post hoc test were used in the statistical analysis

The discrimination index (DI) is the difference between the amount of time spent exploring a novel object and the amount of time spent exploring a familiar object, divided by the total amount of time spent exploring both novel and familiar objects [*DI* = (*T*_N_ − *T*_F_) / (*T*_N_ + *T*_F_)]. Noticeably, the Api-control group was similar to the normal control group in all measured parameters. The DI was significantly decreased by 1.36 folds in the MTX-induction group compared with that in the control group. However, Api co-treatment pre- and post-MTX administration meaningfully increased the DI by 2.12 folds compared with its level in the MTX-alone group (Fig. 2A).

The preference index (PI) was determined by dividing the time spent exploring the novel object by the total time spent investigating both novel and familiar objects, and then multiplying the result by 100. In the MTX group, the PI dropped by 82.9% in comparison with that in the normal control group. However, Api co-treatment significantly raised the PI by 4.4-fold (Fig. 2B).

**Fig. 3:**
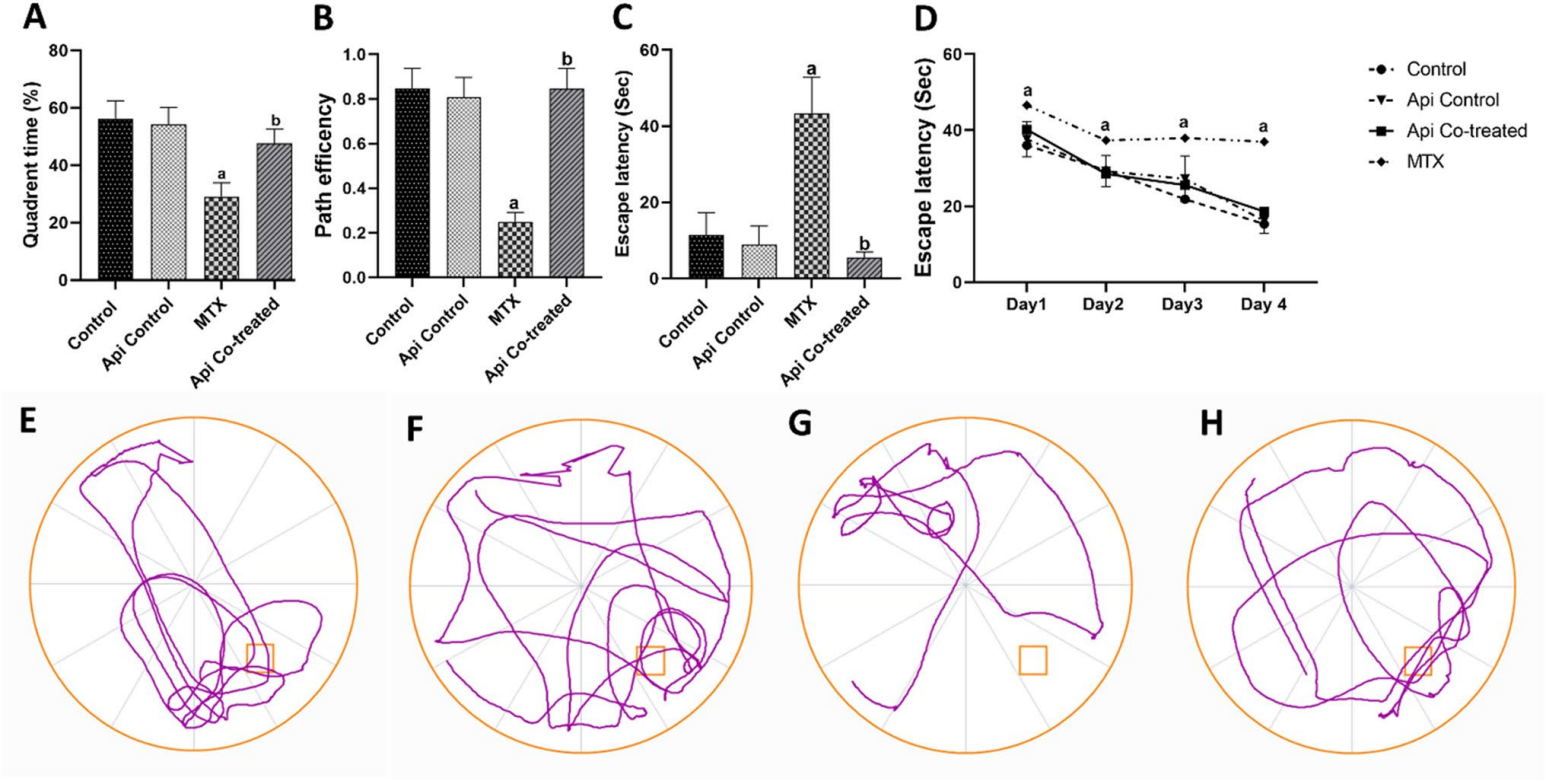
Effects of MTX and Api co-treatment on cognitive function (Morris water maze test). **A** quadrant time, **B** path efficiency, **C** escape latency for probe test, **D** escape latency for training days (days 1–3) and probe test (day 4), **E** track plot for normal control, **F** track plot for Api control, **G** track plot for MTX, and **H** track plot for Api co-treated. Data are expressed as mean ± SD, *n* = 10. (a) significant difference from control, (b) significant difference from MTX. Significance level was set at *P* < 0.05. One-way ANOVA test and Tukey’s post hoc test were used in the statistical analysis, except fot the training and probe escape latency where two-way ANOVA was used

In MWM, we observed that the quadrant time percentage (the proportion of time the rat spent in the target quadrant during the probe test) was 52% lower in the MTX group compared with that in the normal control group. On the other hand, the quadrant time increased by 1.63-fold in the Api co-treated group compared with that in the MTX group (Fig. 3A). The path traveled by the rats was tracked by the ANY-maze software and plotted in the track plots, which clearly indicate that the Api co-treated group spent more time in the quadrants than the MTX group (Fig. 3E–H). In addition, we observed the path efficiency, which is the ratio of the actual path length traveled by a rat to the optimum path it could have taken to reach the target quadrant. The MTX group demonstrated a roughly 60% decline in path accuracy relative to the control group; however, the Api co-treated rats demonstrated a 3.4-fold increase in path efficiency relative to the MTX group (Fig. 3B).

Furthermore, the escape latency was measured to appraise the learning ability of the rats. Rats in the MTX group took 3.79 folds longer time to reach the platform area than normal rats. Api co-treatment throughout the study significantly reverted the learning ability of rats back to the control levels as it showed a noticeable decrease in the escape latency time by 87.24% of that in the MTX group (Fig. 3C). Examining the escape latency during the training trials (days 1–3) and the probe test (day 4) to examine the learning pattern of rats revealed that the MTX group had lower learning ability and the escape latency progress over days than the control and Api co-treated groups (Fig. 3D).

### Api Modulates miR-15a/ROCK-1 in the Hippocampus of MTX-Treated Rats

**Fig. 4:**
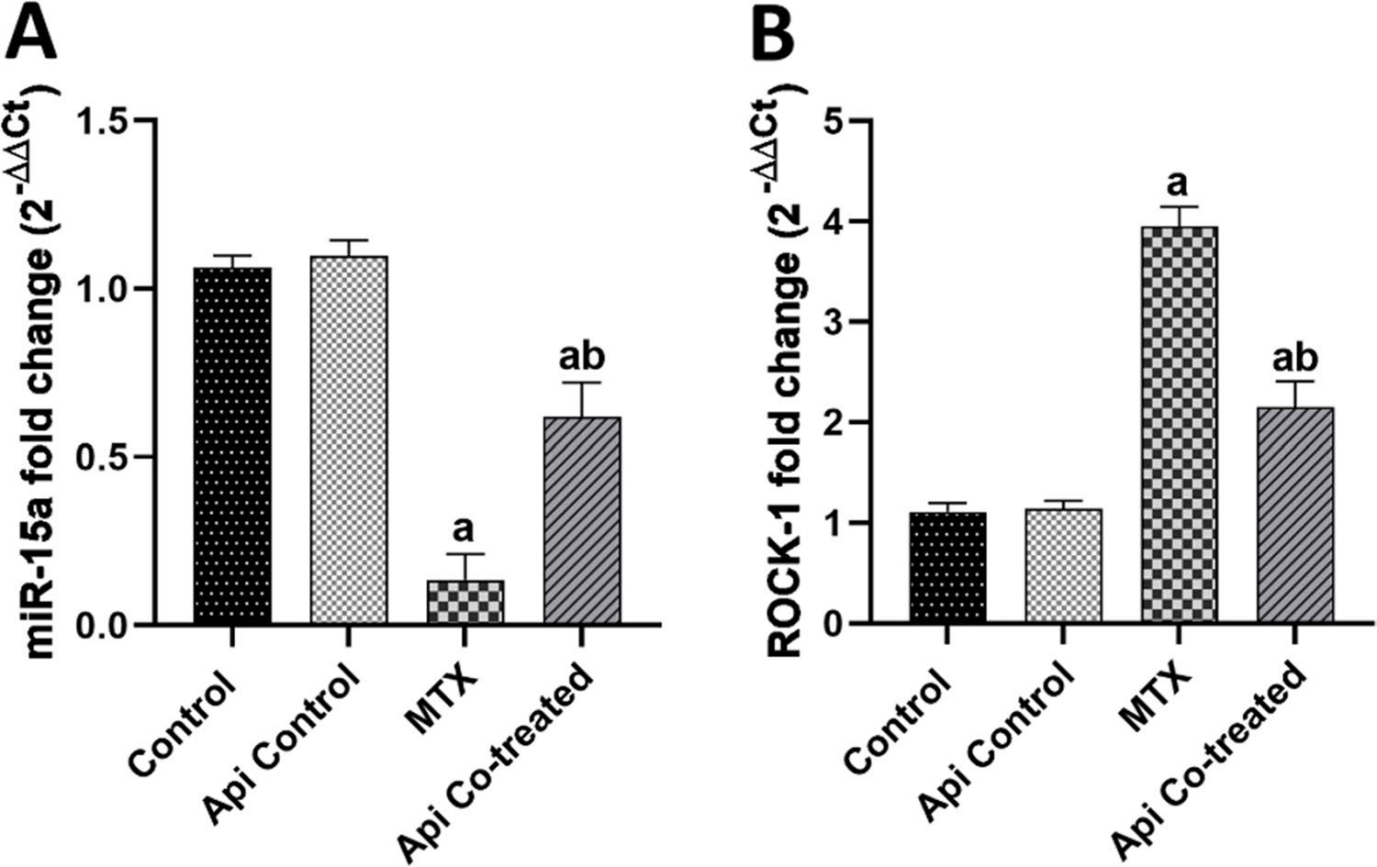
Effects of MTX and Api co-treatment on miR-15a/ROCK-1 levels in the rat hippocampus. Data are expressed as mean ± SD, *n* = 6. (**a**) significant difference from control, (**b**) significant difference from MTX. Significance level was set at *P* < 0.05. One-way ANOVA test and Tukey’s post hoc test were used in the statistical analysis

MTX administration abated the expression of miR-15a in the hippocampus reaching 12.6% of the normal control values. This was accompanied by an increase in the levels of its downstream target ROCK-1 by 3.51 folds in comparison with levels in the control rats (Fig. 4A,B). These results highlighted the neurotoxic effect of MTX on the molecular level.

The Api co-treated group exhibited a significant escalation in the expression levels of miR-15a by 4.63 folds compared with those in the MTX-alone group (Fig. 4A). This increase obviously led to a decline in the ROCK-1 transcription factor level by 54.46% (Fig. 4B), suggesting Api as a possible co-treatment to restore the neuroprotective miR-15a levels.

### Api Mitigates the MTX Effect on the Hippocampal ERK1/2/CREB/BDNF Pathway

**Fig. 5:**
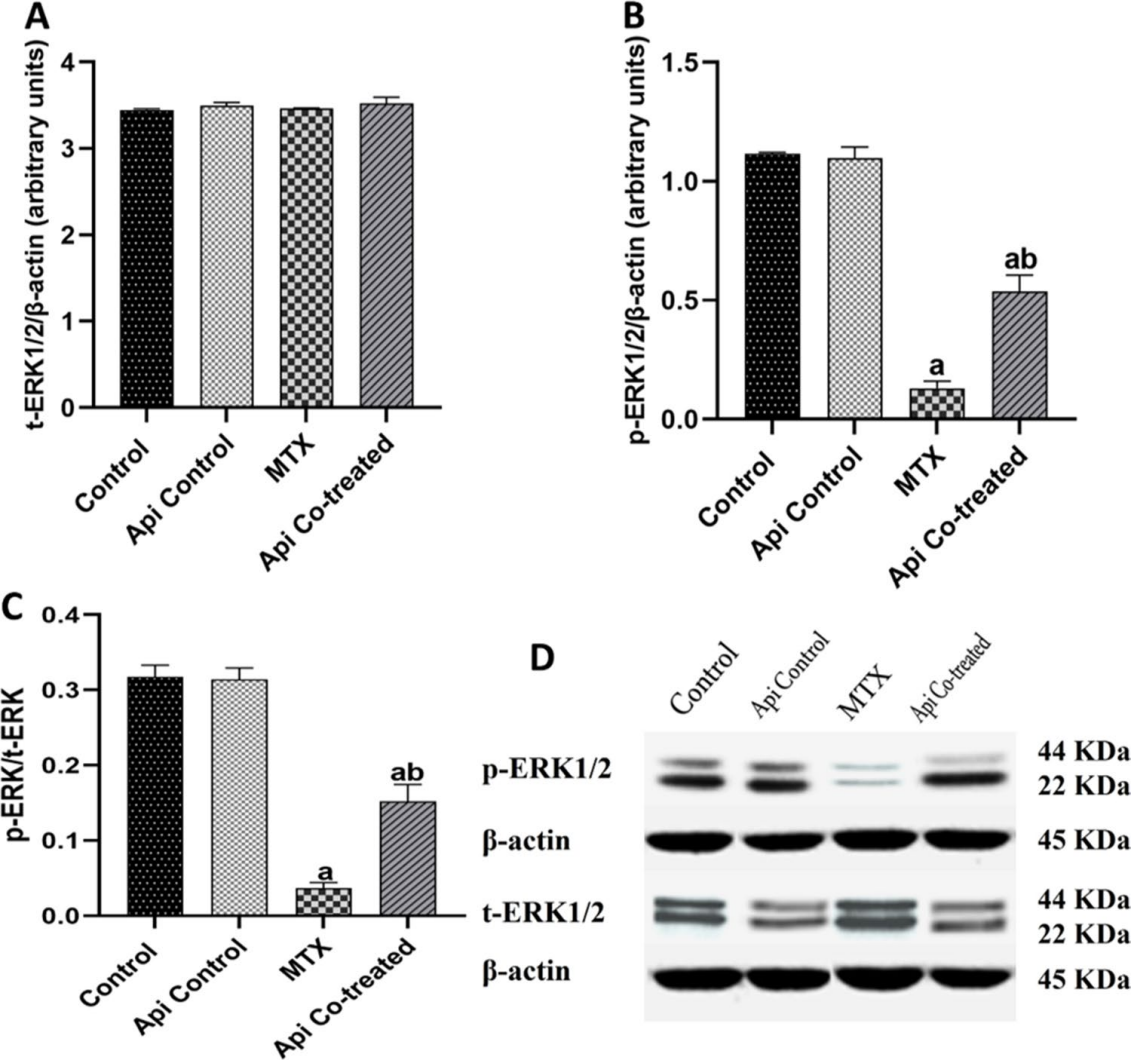
Effects of MTX and Api co-treatment on the ERK1/2 protein expression. Data are expressed as mean ± SD, *n* = 6. (**a**) significant difference from control, (**b**) significant difference from MTX. Significance level was set at *P* < 0.05. One-way ANOVA test and Tukey’s post hoc test were used in the statistical analysis

**Fig. 6:**
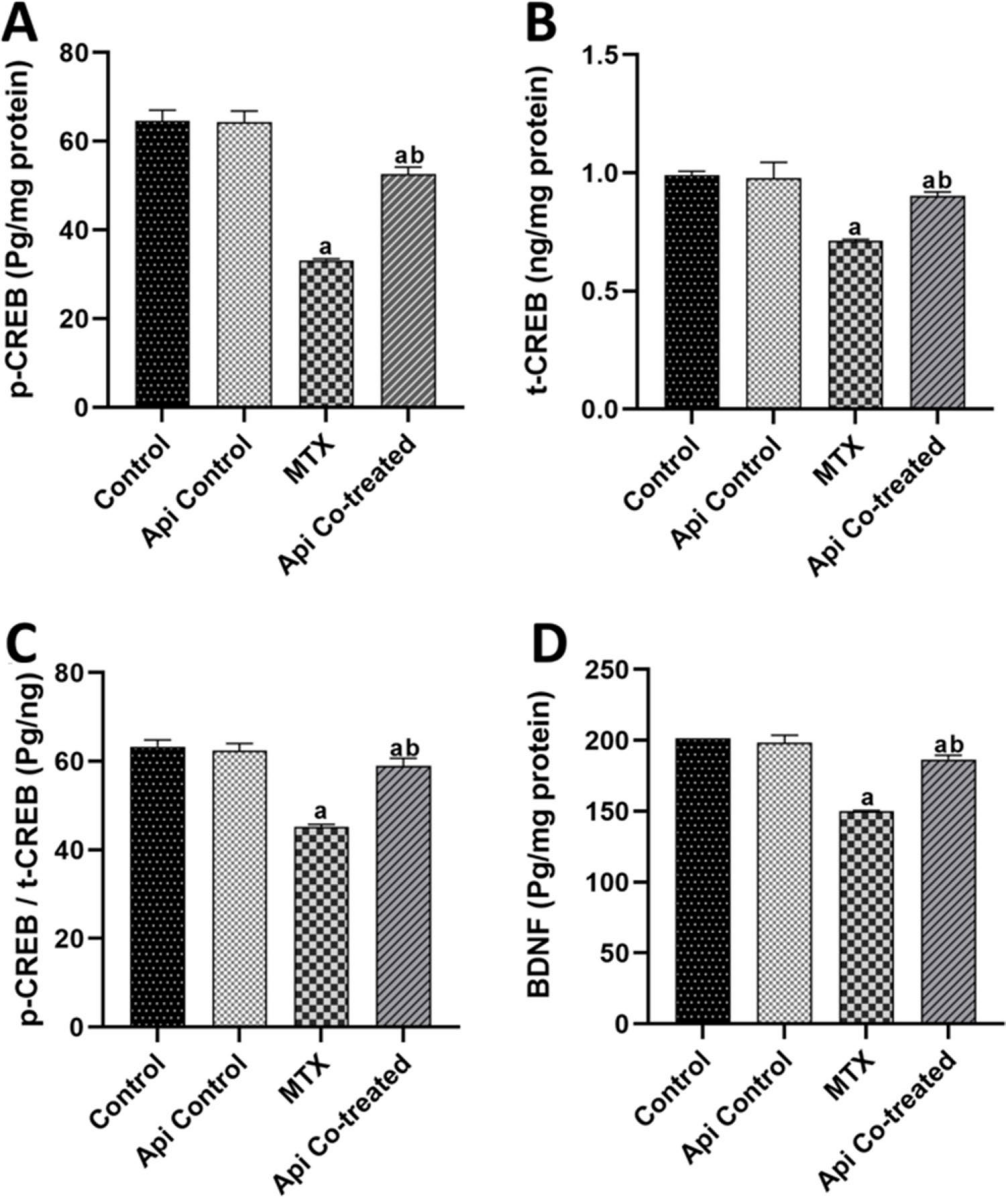
Effects of MTX and Api co-treatment on the CREB/BDNF protein expression. Data are expressed as mean ± SD, *n* = 6. (**a**) significant difference from control, (**b**) significant difference from MTX. Significance level was set at *P* < 0.05. One-way ANOVA test and Tukey’s post hoc test were used in the statistical analysis

In the context of ROCK-1 elevation in the MTX group, the expression of t-ERK1/2 was not affected; however, its activation was reduced. This was marked by the observed downregulation of p-ERK1/2 levels by 88.47% as well as an 8.71-fold reduction in p-ERK/t-ERK ratio in the MTX-treated rats compared with levels in the normal control rats (Fig. 5A–D). Subsequently, p-CREB, t-CREB levels, and p-CREB/t-CREB ratio were recorded to decline in the hippocampus by 48.66%, 28.1%, and 28.64%, respectively, in the MTX group compared with the normal control values (Fig. 6A–C). Moreover, the hippocampal BDNF levels were found to be 25.96% lower in the MTX group than that in the normal control rats (Fig. 6D).

Herein, the neuroprotective effect of Api was set clear by its effect on the ERK1/2/CREB/BDNF pathway. Downstream of ROCK-1, t-ERK1/2 expression was not affected significantly while p-ERK1/2 and p-ERK/t-ERK ratio were significantly elevated in the Api co-treated group by 4.15 and 4.18 folds, respectively (Fig. 5A–D). Consequently, p-CREB, t-CREB levels, and p-CREB/t-CREB ratio were escalated by 1.5, 1.26, and 1.3 folds respectively in the hippocampus of Api co-treated rats compared with those in the MTX-alone group (Fig. 6A–C). Additionally, Api exerted a protective effect, raising the BDNF level to 1.24 times its level in the MTX group (Fig. 6D).

### Api Co-treatment Attenuates the Microglial Activation Induced by MTX in the Rat Hippocampus

**Fig. 7:**
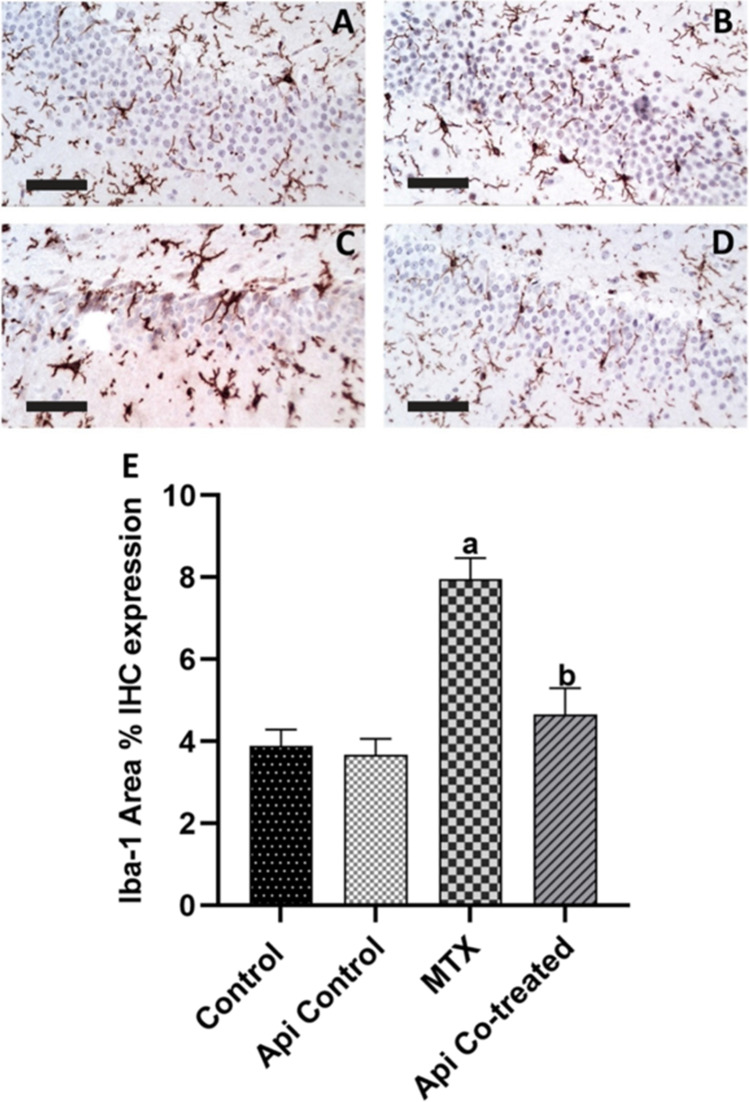
Effect of MTX and Api co-treatment on microglial activation in the rat hippocampus. Data are expressed as mean ± SD, *n* = 4. (**a**) significant difference from control, (**b**) significant difference from MTX. Significance level was set at *P* < 0.05. One-way ANOVA test and Tukey’s post hoc test were used in the statistical analysis. IHC, immunohistochemistry (**A**–**D** anti-Iba-1 immunostaining, 400 ×)

Iba-1 immunohistochemical staining has been considered an indicator for microglial activation. Iba-1 is a particular protein that plays a role in membrane ruffling and phagocytosis in activated microglia [34]. The effect of MTX and Api co-treatment is figured in Fig. 7A–D.

The area % of immunohistochemical expression of Iba-1 was significantly elevated in MTX rats by 2.05 folds compared with that in the normal control rats. On the other hand, administration of Api markedly decreased the Iba-1 expression by 41.63% compared with that in the MTX-alone treated rats (Fig. 7E).

### Api Abolishes the MTX-Induced Oxidative Stress, Neuroinflammation, and Apoptosis in the Rat Hippocampus

**Fig. 8:**
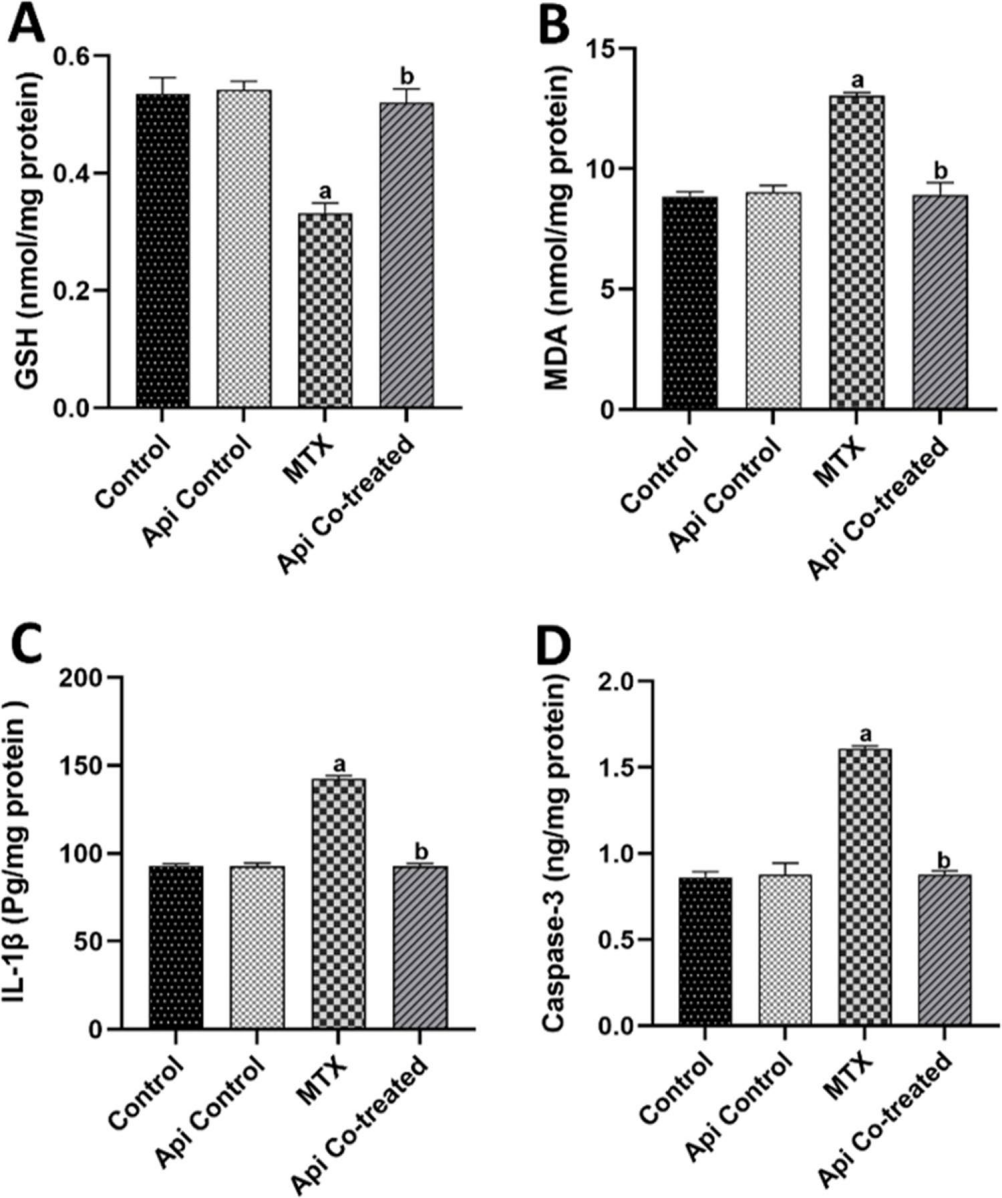
Effects of MTX and Api co-treatment on oxidative stress, neuroinflammation, and apoptosis in the hippocampus. Data are expressed as mean ± SD, *n* = 6. (**a**) significant difference from control, (**b**) significant difference from MTX. Significance level was set at *P* < 0.05. One-way ANOVA test and Tukey’s post hoc test were used in the statistical analysis

Both microglial activation and BDNF decline are known to induce oxidative stress, as evidenced by the recorded reduction of GSH levels by 38.05% along with elevation of MDA levels by 1.47 folds in the hippocampus of MTX-treated rats in comparison with levels in the control rats. Api administration throughout the study normalized the GSH and MDA levels, demonstrating its antioxidant capacity (Fig. 8A,B).

IL-1β levels were observed to increase by 1.53 folds in the hippocampal tissue following MTX treatment compared with those in the control rats. Enhanced apoptosis was also clear in the escalated hippocampal caspase-3 levels, which increased by 1.86 folds when compared with levels in the normal control rats. On the other hand, Api co-treatment counteracted these effects by inhibiting neuroinflammation and apoptosis, as evidenced by normalizing IL-1β and caspase-3 levels (Fig. 8C,D). Together, these data accentuate the anti-inflammatory and antiapoptotic actions of Api.

### Api Protects the Hippocampal DG and CA3 Regions Against Neuronal Degeneration Induced by MTX

**Fig. 9:**
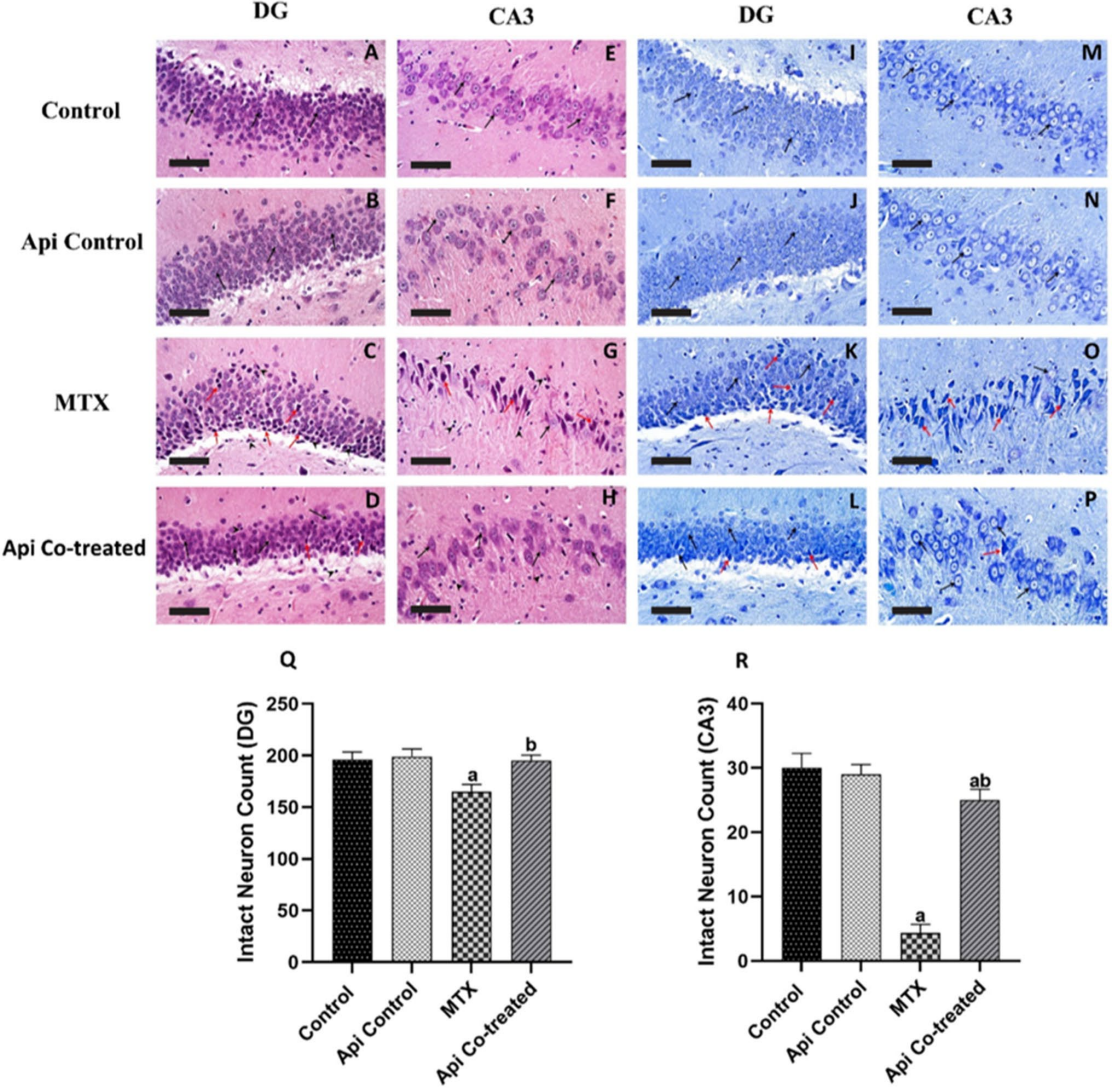
Histopathological effects of MTX and Api co-treatment in the DG and CA3 regions of the hippocampus. **A**–**D** DG H&E stain. **E**–**H** CA3 H&E stain. **A** Normal control and **B** Api control, both demonstrated normal morphological features of hippocampal layers in the DG region, including granule cells at different zones with intact subcellular details (arrow) as well as hilar region without abnormal alterations. **C** MTX treatment increased degenerated granule neurons and nuclear pyknosis (red arrow). Moderate edema accompanied by mild higher reactive glial cells infiltrates (arrowhead). **D** Api co-treatment showed markedly higher neuroprotective effects with several images of intact granule cells with intact subcellular features and increased chromatin condensation (black arrow). Few sporadic degenerated neurons (red arrow) with minor glial infiltration were seen. **E** Normal control and **F** Api control, both demonstrated normal morphological structures of hippocampal layers in CA3 region with apparent intact well-organized pyramidal neurons with intact nuclear and subcellular details (black arrow). Intact intercellular matrix was observed with minimal reactive glial cells infiltration. **G** MTX treated, showed severe neuronal loss and abundant records of hyperesenophilic, angular necrotic pyramidal neurons without distinct subcellular details (red arrow) alternated with few scattered apparent intact cells (black arrow), mild perineuronal edema were shown in brain matrix with marked higher reactive microglial cell infiltrates (arrowhead). **H** Api co-treated demonstrated much superior neuroprotective efficacy with sporadic few deteriorated or necrotic neurons (red arrow) and significantly higher figures of intact well-organized neurons (black arrow). Mild persistent reactive glial cell infiltrates were shown (arrowhead). **I**–**P** Nissl staining of DG and CA3 to detect intact (black arrow) and damaged (red arrow) neurons. **Q** Intact neurons count in DG. **R** Intact neurons count in CA3 area. Data are expressed as mean ± SD, *n* = 4. (a) significant difference from control, (b) significant difference from MTX. Significance level was set at *P* < 0.05. One-way ANOVA test and Tukey’s post hoc test were used in the statistical analysis. **A**–**D** H&E 400 × , **E**–**H** H&E 1000 × , **I**–**L** Nissl staining 400 × , **M**–**P** Nissl staining 1000 ×

Both normal and Api-control rats showed hippocampal layers with intact DG granule cells and a well-organized pyramidal neuron layer in CA3 with preserved nuclear and subcellular characteristics. MTX treatment exacerbated granule neuron degeneration in the DG and neuronal loss and shrinkage in CA3. In addition to intact-looking cells and perineuronal edema, the brain matrix had activated microglial cells. Api co-treated groups had more intact, well-organized granule cells with intact subcellular characteristics and higher chromatin condensation. Co-treatment with Api improved neuroprotective effectiveness, with fewer damaged or necrotic neurons and more intact, well-organized neurons. Mild persistent glial infiltrates were also seen (Fig. 9A–H).

We further employed Nissl staining to detect degraded and intact neuron counts in DG and CA3 from all experimental groups (Fig. 9I–P). The intact neuron count in DG was recorded to decrease by 15.9% in MTX when compared with that in the normal control group. However, Api co-treatment was able to normalize the number of intact neurons (Fig. 9Q). In CA3, the intact neuron count was dramatically reduced in the MTX group in comparison with the decrease that occurred in the DG. Furthermore, Api co-treatment raised the intact neuron count by 5.77 folds compared with that in the MTX group (Fig. 9R).

### Correlation Study

**Fig. 10:**
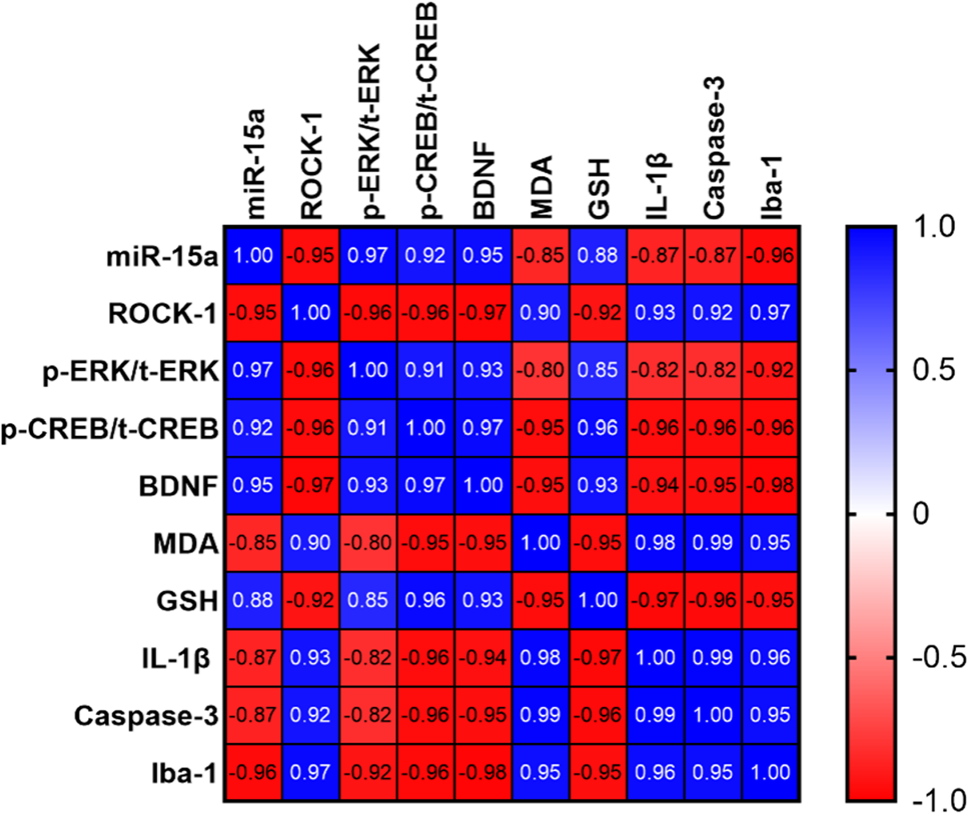
Heatmap of the correlation matrix between different biochemical and immunohistochemical parameters in all studied groups. Values are expressed as the Pearson *r* correlation coefficient, with statistical significance *P* < 0.0001 for all correlations. For each parameter, data from all studied groups were included, *n* = 24, except Iba-1 *n* = 16

Several significant correlations between the various evaluated parameters are depicted in Fig. 10. miR-15a was positively correlated with ERK1/2/CREB/BDNF pathway parameters and GSH, and negatively correlated with ROCK-1, MDA, caspase-3, IL-1β, and Iba-1. ROCK-1 exhibited negative correlations with ERK1/2/CREB/BDNF and GSH, but it exhibited positive correlations with MDA, caspase-3, IL-1β, and Iba-1. All ERK1/2/CREB/BDNF pathway parameters showed similar significant correlation pattern, positively with GSH and negatively with MDA, caspase-3, and IL-1β. The microglial activation marker Iba-1 depicted substantial positive correlations with ROCK-1, MDA, caspase-3, and IL-1β, and significant negative correlations with miR-15a, ERK1/2/CREB/BDNF pathway parameters, and GSH (*P* < 0.0001 for all).

## Discussion

The activation of microglia was one of the most recent hypotheses that were put forward to explain MTX-induced neurotoxicity [35]. To our knowledge, the current study is the first to decipher the molecular underpinnings of MTX-induced microglial activation and to reveal that MTX mechanistically achieves this effect via downregulating miR-15a, raising the gene expression of its reported target ROCK-1, and subsequently decreasing the downstream p-ERK1/2, p-CREB, and BDNF levels in the rat hippocampus. This instigated microglial activation as evidenced by increased Iba-1 immunostaining, ultimately incurring neuroinflammation, oxidative stress, and apoptosis, as shown by increased IL-1β, lipid peroxidation, and caspase-3 levels, respectively. Furthermore, this study is the first to provide evidence that Api mitigated the neurotoxicity of MTX by modulating the miR-15a/ROCK-1/ERK1/2/CREB/BDNF pathway, thereby in part reducing the microglial activation in the rats’ hippocampi. These beneficial effects were concordant with inhibition of neuroinflammation and apoptosis, restoration of the redox balance, and improvement in cognitive function, delineating its neuroprotective effects.

Herein, a considerable rise in Iba-1 immunohistochemical expression in the hippocampus of MTX-treated animals was observed along with increased glial cell infiltrates in the DG and CA3 hippocampal regions, indicating heightened microglial activation. These findings were in line with the growing evidence associating MTX with microglial activation [6, 36, 37]. Recent data highlighted heightened activation of the microglia to be a cause of neurodegenerative diseases including AD, parkinsonism, and frontotemporal dementia [38–40]. Microglial stimulation is known to create reactive astrocytes that affect the normal function of neurons and is thought to be involved in neurodegenerative disorders [41–43]. The microglia are CNS-resident macrophages, and it has been demonstrated that microglia with both M1 and M2 phenotypes play a dual role in most neurodegenerative diseases. M1 is a proinflammatory cell that induces the generation of reactive oxygen species and IL-1β, whereas M2 is an anti-inflammatory cell [44]. To note, MTX was shown to induce chronic microglial activation and subsequent derangements that were dependent on the inflammatory microglia [6]. Our data are in line with M1 phenotype activation, confirmed by the observed histopathological alterations and the neuroinflammatory status evidenced by elevated levels of IL-1β in the hippocampus of MTX-treated rats.

In this study, we elaborated on the molecular mechanism underlying the microglial activation induced by MTX. Given that the link between ROCK-1 induction and microglial activation was reported [45], we explored the effect of MTX on ROCK-1 as a possible mediator and further expatiated its upstream and downstream effectors. Our data revealed upregulation of ROCK-1 along with downregulation of its upstream effector miR-15a and reduction of its downstream target ERK1/2 in the hippocampi of rats treated with MTX. Indeed, previous data has associated ROCK-1 upregulation with various neurodegenerative disorders, including AD [12, 46]. Furthermore, miR-15a downregulation, upstream of ROCK-1, was previously associated with AD [47]. Herein, miR-15a was dramatically downregulated in the hippocampus of MTX-treated rats, supporting the previous findings which correlated the downregulation of miR-15a with the overexpression of ROCK-1 [18, 48]. ROCK-1 activation has been lately proven to have a role in mediating neuroinflammation via directly evoking microglial activation through increasing M1 neuroinflammatory phenotype size and count [49, 50] or indirectly via the effect of ROCK-1 on ERK1/2/CREB/BDNF pathway [51, 52]. This was proven by the observed negative correlations between ROCK-1 and miR-15a as well as ERK1/2/CREB/BDNF pathway parameters.

ROCK-1 has been proven to negatively affect ERK1/2 activation [53]. Notably, we found a significant decline of activated ERK (p-ERK1/2) manifested by the reduced p-ERK/t-ERK ratio in the hippocampus of the MTX-alone group. In our study, p-ERK1/2 showed a direct relation with CREB phosphorylation, with a decline in activated CREB (p-CREB) as well as p-CREB/t-CREB ratio was noticed in the hippocampus of the MTX-treated group. We recorded a significant decline in BDNF levels downstream of CREB due to MTX injections. These results support the previous data reporting that decreased CREB/BDNF axis results in impaired neurogenesis and neuroinflammation as well as neuronal survival [16, 48, 54].

Interestingly, microglial activation was shown to perpetuate the decline in BDNF levels, resulting in cognitive impairment [8]. Indeed, microglial activation invokes an imbalance between proinflammatory and anti-inflammatory cytokines and prompts a reduction in cell proliferation and BDNF levels, leading to activation of apoptosis, reduced hippocampal neurogenesis, and spatial memory loss [7]. BDNF is one of the most important neurotrophins regulating brain homeostasis. Hippocampal BDNF enhances neuronal survival, neuroplasticity, and neurogenesis, preserving learning, memory, and cognitive functions of the brain. BDNF binds to the tyrosine kinase B receptor (TrkB) activating neuroprotective pathways, including the ERK1/2/CREB pathway, which itself upregulates BDNF [40, 55].

Compelling data suggest that microglial activation as well as decreased CREB/BDNF to be important activators for oxidative stress and inflammatory cytokines in AD, autism, and neurodegenerative disease, leading to cognitive and learning impairment [7, 56–58]. Moreover, earlier research has linked microglial activation, IL-1β elevation, and BDNF reduction to anesthesia- and surgery-induced hippocampus cognitive impairment [59]. In this context, MTX was found to have neuroinflammatory side effects through provoking oxidative stress and a proinflammatory milieu. Eliciting oxidative stress by MTX in the rat hippocampi led to an increase in the inflammatory mediator IL-1β and the apoptotic marker caspase-3. This was evidenced by the observed decrease in GSH levels, elevation of lipid peroxidation, and a marked increase in IL-1β levels in the hippocampus. A mechanistic explanation of the increased neuroinflammation could be based on ROCK-1-mediated microglial activation through an altered miR-15a/ROCK-1/ERK1/2/CREB/BDNF pathway.

Our findings from the correlation analysis implied elevated expression of ROCK-1 to increase oxidative stress and proinflammatory markers, leading to activated apoptosis and neural death. This supports the data of a recent study that has associated ROCK-1 activation with increased inflammatory cytokines in the hippocampus, which leads to neuronal death and cognitive dysfunction [10]. Caspase-3 which is an important protease in the apoptosis process was found to increase in the MTX group, which indicates that MTX is a neuro-lethal drug. This data was also supported by the histopathological examination of the hippocampus, which revealed higher records of degenerated granule neurons in the MTX group hippocampi. Furthermore, Nissl staining showed a significant decrease in intact neurons in both DG and CA3. Therefore, our results spotlight that both activation of microglia and downregulation of BDNF by MTX are possible mechanisms that underlie the neuroinflammatory apoptotic consequences.

Our behavioral analysis data showed a significant decline in the learning ability and cognitive function due to MTX treatment. These data came in line with recently reported data linking MTX administration to an increased risk of cognitive impairment and memory problems in juvenile patients, resulting in an impaired learning outcome [60]. In the performed NOR test, the MTX group showed a negative discrimination index and lower preference index, which means that the rats were totally unable to discriminate between old and novel objects during the choice trial. The conducted MWM test came up with a similar observation, which revealed a dramatic elevation in the escaping latency of the MTX-treated rats in addition to a substantial decrease in both the quadrant time and path efficiency of the probe test. Our data support previous reports suggesting a negative correlation between BDNF levels and escape latency [61] as well as elevation of escape latency in microglial activation models [62].

Our work further contemplated the neuroprotective effect of Api on MTX-induced neurodegeneration. Recent growing data highlighted Api as a neuroprotective naturally occurring flavonoid in parkinsonism [63], methylmercury neurotoxicity [64], and depressive disorders [65]. In particular, Api was studied for its neuroprotective action in various models due to its antioxidant and anti-inflammatory activity [65, 66]. In our study, Api co-treatment proved itself as a potential antidote for MTX-induced neuroinflammation, attenuating its neurotoxicity. The Api co-treated group showed a 4.63-fold elevation in the gene expression of miR-15a and a substantial reduction in ROCK-1 expression. Consequently, microglial activation was mitigated as shown by the substantial decline in Iba-1 levels in the hippocampus of the Api co-treated group, reaching 58.37% of levels in the MTX-alone group. Similarly, a previous study demonstrated that Api inhibits M1 microglia [65]. Furthermore, Api has been reported to have an inhibitory action on microglial inflammatory effects in cultured microglia and in in vitro models of neuroinflammation associated with AD [21, 67]. Moreover, recent data highlighted the indirect inhibitory effect of Api on ROCK-1 expression in hepatocellular carcinoma models in vivo and in vitro [68] as well as the upregulatory effect on BDNF in kindled mice models [19]. Taken together, these data support that ROCK-1 inhibition by Api could be neuroprotective against MTX-induced derangements in the rat brain.

Our results further elucidated the molecular mechanisms of the Api neuroprotective effects of ROCK-1 inhibition. Api seems to activate the ERK1/2/CREB/BDNF pathway, attested by elevated p-ERK1/2, p-CREB, and BDNF levels in the rats’ hippocampi. Concurrently, elevation of GSH and decrements in lipid peroxidation, IL-1β, and caspase-3 levels were proven by our analysis of inflammatory, oxidative, and apoptotic markers in the hippocampus of Api co-treated rats. These results are in alignment with previously published data that Api reduced oxidative stress and inhibited apoptosis in various models, including doxorubicin-induced cardiotoxicity [69] and parkinsonism [63]. Furthermore, previous studies demonstrated similar results for Api on GSH, MDA, and IL-1β [70]. Thus, one can speculate that both inhibition of microglial activation and ERK1/2/CREB/BDNF pathway activation potentiate the anti-inflammatory, antioxidant, and antiapoptotic effects of Api in this model. These protective effects explain the increased neuronal survival and neuroplasticity in the hippocampi of Api co-treated rats, showing higher intact neuron count in both DG and CA3 regions. This was reflected in the substantial improvement in cognitive and learning behavior manifested by increments in the discriminating index in NOR and the escape delay in MWM.

Our findings emphasized the impact of ROCK-1 inhibition as a possible target for attenuating MTX-induced neuroinflammation. This was consistent with previous studies that highlighted ROCK-1 inhibition as an important player in neuroprotection and inhibiting neuroinflammation via multiple mechanisms, such as decreasing both microglial activation and oxidative stress by shifting between M1 and M2 microglia phenotypes, inhibiting apoptosis [12], and its effect on activating the ERK1/2/CREB/BDNF neuroprotective pathway [51]. Api suppressed ROCK-1, reduced microglial activation, neuroinflammation, oxidative stress, and apoptosis, establishing itself as a promising co-treatment for mitigating MTX-induced neurotoxicity. This work has noteworthy limitations, and we urge that future research focus on studying the effect of different MTX and Api doses. Furthermore, research potential miRNAs and other neurotrophic factors that may be influenced by the pathways investigated in this study.

## Conclusion

**Fig. 11:**
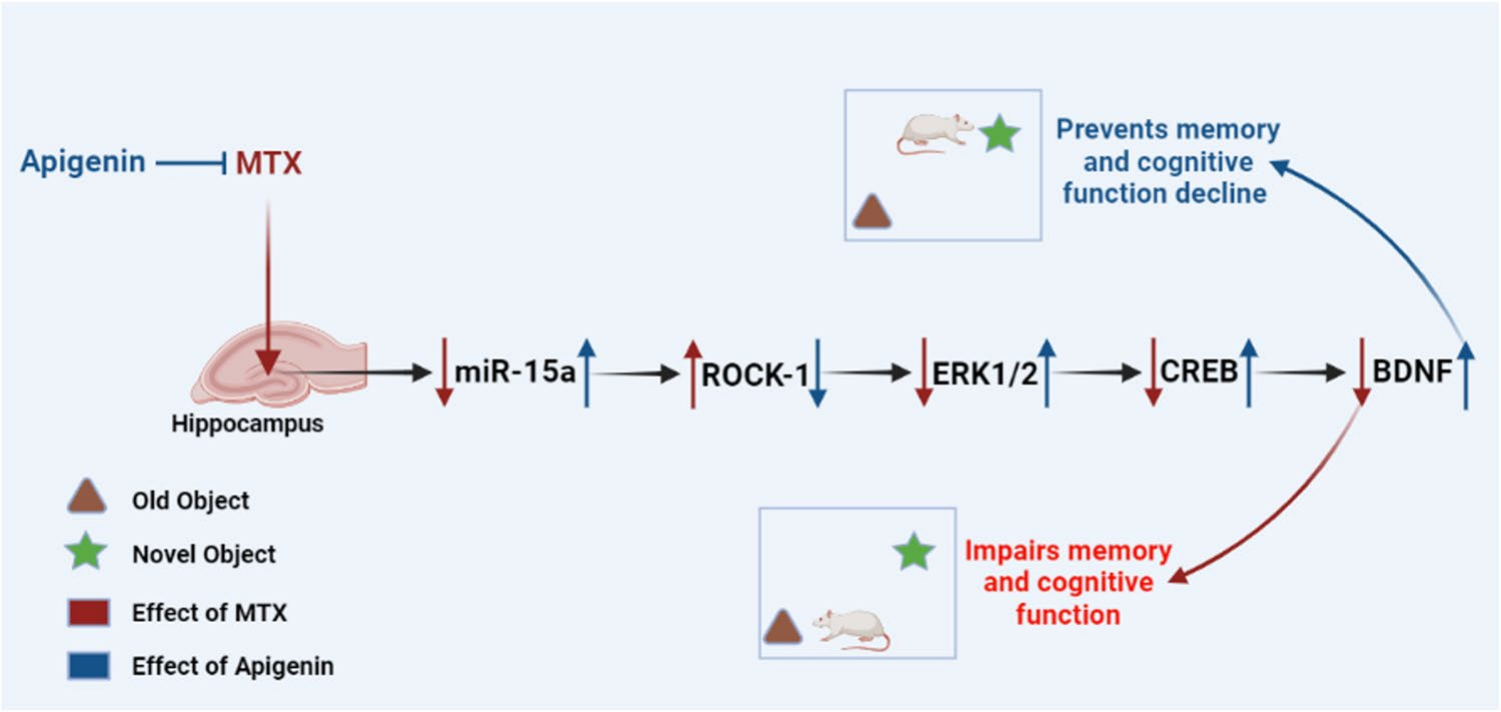
Summative illustrative graph for the study results. MTX administration resulted in the downregulation of hippocampal miR-15a, which induced an increase in the expression of its target ROCK-1, hence blocking the downstream ERK1/2/CREB/BDNF pathway and causing memory loss and cognitive impairment. Api co-treatment, on the other hand, mitigated the MTX effects by restoring miR-15a, reducing ROCK-1 expression, and activating the ERK1/2/CREB/BDNF pathway, resulting in enhanced memory and cognitive function in MTX-treated rats

MTX-induced neurotoxicity and cognitive impairment could be attributed to the microglial activation and neuroinflammatory response in part through the miR-15a/ROCK-1/ERK1/2/CREB/BDNF cascade. This study advocates the neuroprotective effects of Api through its inhibitory effect on MTX-induced microglial activation, along with lessening neuroinflammation, oxidative stress, and apoptosis, and boosting the cognitive function of rats via possible modulation of the miR-15a/ROCK-1/ERK1/2/CREB/BDNF pathway in the hippocampus. Our findings lay the groundwork that Api could serve as a considerable option for attenuating MTX-induced neuroinflammation and neurotoxicity. A summary of the current results is presented in Fig. 11.

## Data Availability

The datasets used and analyzed during the current study are available upon reasonable request from the corresponding author.

## Abbreviations

ALL: Acute lymphoblastic leukemia
AD: Alzheimer’s disease
Api: Apigenin
BDNF: Brain-derived neurotrophic factor
CA: Cornu ammonis
CMC: Carboxymethyl cellulose
CREB: cAMP response element-binding protein
DG: Dentate gyrus
DI: Discrimination index
ELISA: Enzyme-linked immunosorbent assay
ERK1/2: Extracellular signal-regulated kinase 1/2
GSH: Reduced glutathione
Iba-1: Ionized calcium-binding adaptor protein-1
IHC: Immunohistochemistry
IL-1β: Interleukin-1β
LCV: Leucovorin
MDA: Malondialdehyde
miR-15a: MicroRNA-15a
MTX: Methotrexate
MWM: Morris water maze
NOR: Novel object recognition
PI: Preference index
ROCK-1: Rho-associated protein kinase-1
RT-qPCR: Reverse transcriptase-quantitative polymerase chain reaction
SD: Standard deviation
